# Leukocyte telomere length and telomerase activity in Long COVID patients from Rio de Janeiro, Brazil

**DOI:** 10.1590/0074-02760240129

**Published:** 2025-04-11

**Authors:** Giane Gonçalves dos Reis, Rafaele Tavares Silvestre, Gilda Alves, Lucas Delmonico, Mariana Chantre-Justino, Aline dos Santos Moreira, Beatriz de Lima Alessio Müller, Carolina Ramos do Nascimento, Denzel Luis Pereira da Silva, Louisy Sanches dos Santos, Ana Luíza de Mattos-Guaraldi, Maria Helena Ornellas

**Affiliations:** 1 Universidade do Estado do Rio de Janeiro, Faculdade de Ciências Médicas, Laboratório de Marcadores Circulantes, Rio de Janeiro, RJ, Brasil; 2 Universidade do Estado do Rio de Janeiro, Faculdade de Ciências Médicas, Pós-Graduação em Ciências Médicas, Rio de Janeiro, RJ, Brasil; 3 Universidade do Estado do Rio de Janeiro, Faculdade de Ciências Médicas, Departamento de Microbiologia, Imunologia e Parasitologia, Rio de Janeiro, RJ, Brasil; 4 Instituto Nacional de Traumatologia e Ortopedia, Rio de Janeiro, RJ, Brasil; 5 Fundação Oswaldo Cruz-Fiocruz, Instituto Oswaldo Cruz, Laboratório de Genômica Aplicada e Bioinovações, Rio de Janeiro, RJ, Brasil; 6 Fundação Oswaldo Cruz-Fiocruz, Instituto Oswaldo Cruz, Rede de Plataformas Tecnológicas Fiocruz, Plataforma de Sequenciamento de Nova Geração, Rio de Janeiro, RJ, Brasil

**Keywords:** post-acute COVID-19 syndrome, post-acute sequelae of SARS-CoV-2 infection, telomere elongation, telomerase, SARS-CoV2

## Abstract

**BACKGROUND:**

Coronavirus disease 2019 (COVID-19) is caused by the new coronavirus 2 (severe acute respiratory syndrome coronavirus 2 - SARS-CoV-2). Long COVID is a new condition associated with persistent COVID-19 symptoms and/or new emerging symptoms. Telomeres are specialised structures for genome protection at the end of chromosomes and telomerase is the enzyme that synthesises telomere DNA.

**OBJECTIVES:**

Patients with Long COVID symptoms were recruited at the Pedro Ernesto University Hospital (HUPE) in Rio de Janeiro, Brazil, with the main purpose of investigating the association between telomere length and Long COVID.

**METHODS:**

Leukocyte telomere length (LTL) was determined by quantitative real-time polymerase chain reaction (qPCR) in 34 Long COVID patients compared to a control group (n = 122). Telomerase activity was determined by qPCR assays using the commercial kit from ScienCell. A questionnaire on symptoms, vaccine doses and blood count was completed.

**FINDINGS:**

The Long COVID patients were found to have an increase in LTL. Telomerase activity was also examined in a smaller number of patients and found to be reactivated in the blood.

**MAIN CONCLUSIONS:**

It will be necessary to conduct further studies and monitor Long COVID patients to determine if future health issues could be linked to telomerase activity and elongated telomeres.

In March 2020, the World Health Organization (WHO) declared the coronavirus disease 2019 (COVID-19) pandemic caused by the new coronavirus 2 (severe acute respiratory syndrome coronavirus 2 - SARS-CoV-2).^1^ SARS-CoV-2 belongs to the family Coronaviridae. This family is part of the order Nidovirales and includes viruses that are enveloped and have a single-stranded RNA genome with positive polarity. Within the Coronaviridae family, SARS-CoV-2 is classified under the genus Betacoronavirus.^2^ ^,^ ^3^ In Brazil alone, COVID-19 was the cause of death of over 700,000 people.^3^ The main risk factor for death was age, although not exclusively. Although the COVID emergency phase lasted until May 2023, 594,036 new cases were reported to the Brazilian Ministry of Health from January to April 2024, posing a serious public health issue.^4^ ^,^ ^5^

After the acute phase of COVID-19, millions of patients around the world reported that COVID-19 symptoms persisted when the virus was no longer detectable, and that new symptoms were appearing. This wide combination of symptoms corresponds to a new condition, Long COVID, also known as post-COVID or post-acute sequelae of COVID-19/SARS-CoV-2 infection (PASC). The symptoms of Long COVID are complex and variable.^6^ ^,^ ^7^ Symptoms may alternate over time with the co-occurrence of other illnesses, and their duration is also variable.^6^ ^,^ ^7^ ^,^ ^8^

Telomeres are specialised protective structures at the end of chromosomes that contain short repetitive sequences and proteins. Telomere length (TL) is shortened naturally by aging processes and in age-related diseases and can be influenced by lifestyle.^9^ ^,^ ^10^ ^,^ ^11^ Nevertheless, telomere dysfunction can also lead to telomere elongation.^9^ Telomerase is the complex enzyme that synthesises telomeric DNA based on an RNA template and the catalytic subunit hTERT.^12^ It is active in rapidly growing cells and is therefore associated with stem cells and cancer.^13^ ^,^ ^14^

TL has been studied in patients diagnosed with acute COVID-19 disease. Shortening and aging of telomeres was related to disease severity.^15^ ^,^ ^16^ ^,^ ^17^ ^,^ ^18^ By contrast, the association between TL and complex Long COVID syndrome has not been fully demonstrated. In this article, we report on LTL examined in 34 Long COVID patients compared to a control group (n = 122). Telomerase activity was also examined in a smaller number of patients (n = 4) to confirm the data.

## SUBJECTS AND METHODS

*Patients and controls* - Patients with Long COVID symptoms were recruited from the outpatient clinic at the Pedro Ernesto University Hospital (HUPE) in Rio de Janeiro, Brazil, from 2021 to 2022. Peripheral blood samples were collected during the same period for regular blood tests and to establish the LTL protocol. Patients were asked about their acute COVID-19 symptoms, hospitalisations and treatments, Long COVID symptoms, COVID-19 vaccine doses administered and previous chronic medical conditions. The 42 symptoms [supplementary data (Table I)] evaluated in this study are consistent with those reported by the WHO and other publications on the subject.^6^ ^,^ ^7^ ^,^ ^19^ ^,^ ^20^ ^,^ ^21^ ^,^ ^22^ The criteria for determining the severity of COVID-19 were based on whether or not the patients were hospitalised. The cases that were hospitalised were classified as severe, while the outpatients were classified as less severe. A total of 30 women and four men (n = 34) with a mean age of 50.6 years [standard deviation (SD) ± 10.7] were recruited.

**Fig. 1:**
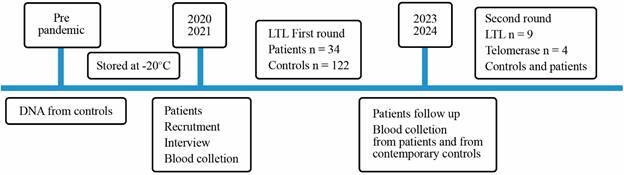
timeline of the leukocyte telomere length (LTL) and telomerase assays. The timeline is not in scale.

The control group for the LTL test consisted of 97 women and 25 men (n = 122) with a mean age of 50.9 years (SD ± 10.5). The age range of the patients and the control group was between 40 and 60 years. This age range was applied, as aging may not be a linear process, with dysregulations in the immune system and metabolism were detected at around 44 years and 60 years, respectively.^23^ The DNA of the control group was prepared in our laboratory before the COVID-19 pandemic and stored at -20ºC. In this way, any influence of the SARS-CoV-2 infection on genome/telomere instability was excluded. The participants in the control group were recruited in Rio de Janeiro and stated that they had no serious or metabolic diseases. Fig. 1 shows the schedule of blood collection and LTL and telomerase assays, which were performed as follows.

**Fig. 2:**
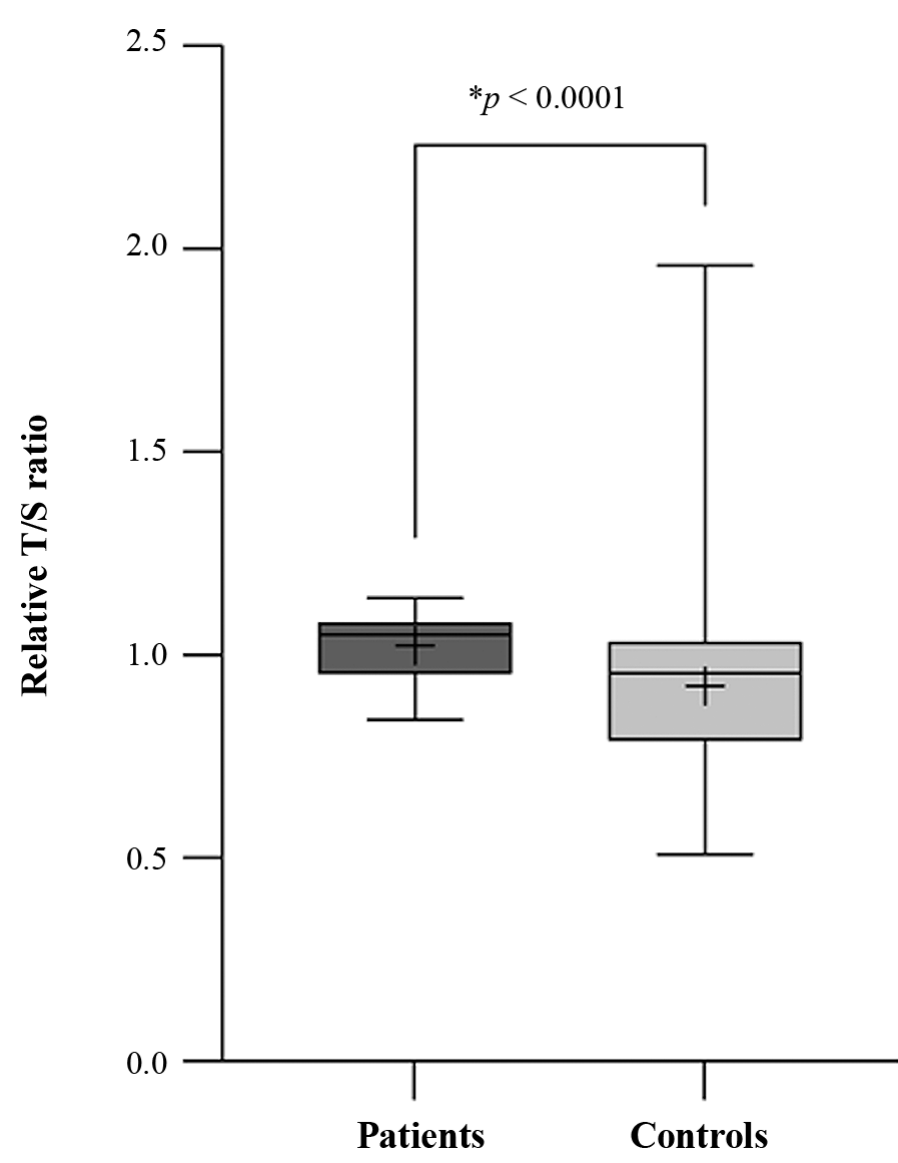
telomere length (T/S) of Long COVID patients x controls in the first round. T/S ratio: ratio of telomere repeat copy number (T) to single copy gene (S); patients n = 34; controls n = 122; p < 0.05 is significant.

*Leukocytes telomere length* - DNA was extracted from peripheral blood mononuclear cells (PBMCs) using the conventional phenol-chloroform method and quantified using the Qubit dsDNA HS Assay Kit (Invitrogen, Waltham, MA, USA) according to the manufacturer’s protocol. LTL was measured using the Cawthon-developed quantitative real-time polymerase chain reaction (qPCR) method, which uses 20 ng of DNA and determines the ratio of telomere repeat copy number (T) to single copy gene (S), T/S.^24^ The single copy gene used in this study was human β-globin (Hbg). The cycling conditions, primers, and reagents used in this study have been previously described.^25^ The PCR runs were duplicated on a StepOnePlus Real-Time PCR System (Applied Biosystems). An example of amplification curves can be seen in Supplementary data (Fig. 1). After each reaction, the melting curves of the products were observed and in all cases the curves (T and S) were in the correct position, indicating that no secondary product was present due to DNA decomposition [Supplementary data (Fig. 2)].

*Telomerase activity* - ScienCell Telomerase Activity Quantification qPCR Assay Kit (catalogue no. 8928) was used according to the manufacturer’s instructions. PBMCs were isolated from 5 mL of a small number of participants: patients (n = 4) and controls (n = 4) who matched for age and sex (all women). Controls were selected from healthy volunteers, as the telomerase assay requires fresh preparation. Two of the control subjects reported no COVID symptoms and tested negative for COVID; one of the control subjects tested positive for COVID but was asymptomatic (over a year ago), and another tested positive but had mild COVID symptoms (over a year ago). According to the protocol, a cycle threshold (Ct) > 33 is considered undetectable for telomerase activity, while Ct < 33 corresponds to positive telomerase activity.

*Statistical analysis* - Contingency tables were used to assess the association between telomere shortening and age, symptom severity, and the distribution of various related symptoms. The statistical significance of the association between these variables was evaluated using the χ2 test and the Fisher exact test. The statistical significance of the association when comparing two or more variables, e.g. different types of symptoms and hospitalisations by severity, was evaluated using the t-test and the Mann-Whitney test.

The non-parametric Kruskal-Wallis test was used to evaluate the significance of the difference between telomere lengths of the studied groups. Linear regression was used to assess the correlation between age and telomere length between the groups studied. The survey data were processed in GraphPad version 9.4.1. A significance level of 5% was considered for all statistical tests. Thus, statistically significant relationships were considered to be those whose p-value was < 0.05.

*Ethics* - This work was approved by the Pedro Ernesto Ethics Committee under number CAAE 50511821.8.0000.5259.

## RESULTS

*Clinical characteristics of the patients* - During the acute phase of infection, 10 patients (29.4%) were hospitalised due to the severity of their condition. Hospitalisation occurred between November 2021 and December 2022. The average length of hospitalisation was 15 days. Based on diagnostic data, patients were infected with either the Delta, Gamma or Omicron variant.^26^ ^,^ ^27^ COVID symptoms appeared to be the consequence of the acute infection (runny nose, sore throat, back pain, fatigue, etc.). On the other hand, the most frequent long-term COVID symptoms reported by patients were fatigue (58.8%), shortness of breath (52.9%) and body aches (50%) [Supplementary data (Table I)]. Regarding chronic conditions before COVID-19, hypertension was the most common (15/34), followed by respiratory conditions (asthma, rhinitis, sinusitis, bronchitis) (11/34) and diabetes mellitus (8/34).

A follow-up examination was carried out by April 2024 (on average 34.5 months later). Twenty patients (58.8%) remained symptomatic, four (11.8%) were asymptomatic and 10 (29.4%) did not respond to our contact attempts. Fatigue (54.16%) and shortness of breath (54.16%) remained the most common symptoms, along with memory disorders (37.5%). Patients with symptoms continue to be treated by physiotherapy, psychology and various medical specialties (pulmonologists, cardiologists and neurologists). Bronchodilators and antidepressants were the most commonly prescribed medications. Regarding COVID-19 vaccination, 31 patients reported having received at least two doses of vaccine. The number of booster vaccinations decreased over time, with only 10 patients reporting that they had received five doses.

Lymphopenia and an elevated neutrophil/lymphocyte ratio (NLR) have been described as predictors of the severity of COVID-19 infection.^28^ ^,^ ^29^ ^,^ ^30^ Among the Long COVID patients, NLR was observed in only 3/34 [Supplementary data (Table II)]. Only one patient had absolute lymphopenia. Therefore, no association could be established between Long COVID and NLR or lymphopenia. Neutrophilia was observed in four patients.

*Telomere and telomerase* - In the statistical comparison, it was found that the LTL of Long COVID patients was prolonged compared to the control group (p < 0.0001) (Fig. 2). To confirm that telomeres were longer in the Long COVID group, the LTL test was repeated 15 months later in nine patients. The telomere length (T/S) of the 9-patient subgroup was 1.09 (average) in the first round and 1.07 (average) in the second round. The shortening of telomeres observed between the first and second rounds was expected due to aging; however, in the second round, telomeres were still longer on average than those of the control group, confirming the results of the first round. Since Long COVID telomeres were certainly elongated, one hypothesis that could explain this result could be that telomerase was active and continuously adding DNA sequences. Subsequently, telomerase activity was examined in the blood of four female patients and four matching control subjects. Telomerase activity was positive (Ct < 33) in the Long COVID patients and negative (Ct > 33) in the controls, as shown in Table. Using the formula of the ∆∆Ct method, telomerase was 9.85 times more active in the Long COVID patient group than in the control groups.

**TABLE:** Telomerase activity and telomere length (T/S)

Long COVID	Ct	Controls	Ct
1	28.38	1	31.03
2	30.71	2	34.62
3	28.59	3	32.98
4	29.94	4	32.22
Ct average	29.41	Ct average	32.7
Age average	46.3	Age average	46.1
T/S average	1.07	T/S average	1.01

T/S ratio: ratio of telomere repeat copy number (T) to single copy gene (S); Ct: cycle threshold.

**Fig. 3:**
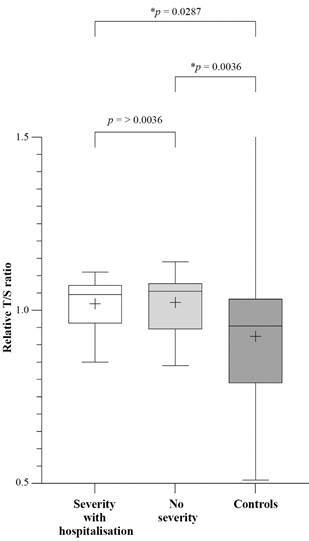
telomere length (T/S) of Long COVID patients according to hospitalisation x controls. T/S ratio: ratio of telomere repeat copy number (T) to single copy gene (S); hospitalised patients n = 10; no severity patients n = 24; p < 0.05 is significant.

The significant telomere elongation observed in Long COVID patients could not be linked to the severity of COVID-19, *i.e.*, the need for hospitalisation. In both the hospitalised and non-hospitalised subgroups, telomeres were found to be significantly elongated, p = 0.0287 and p = 0.036, respectively (Fig. 3).

## DISCUSSION

The diagnosis of COVID-19 was made quickly through a combination of clinical evaluation, testing for the presence of the virus (RT-PCR, antigen or serological), and imaging (chest X-rays and CT scans). By contrast, the diagnosis of Long COVID is in a grey area. So far, no Long COVID biomarker has been discovered in the blood that could help with a quick and accurate diagnosis, making this condition a medical challenge.^31^ Currently, diagnosis is based on a combination of a wide range of clinical symptoms.^20^ One possible explanation for Long COVID could be the remaining SARS-CoV-2 antigens, including the S1 subunit of spike, full-length spike and nucleocapsid, in plasma and serum samples collected months after the end of the acute phase.^32^ These virus particles could stimulate the enhanced expression of inflammatory cytokines.

In this study, 42 persistent symptoms of Long COVID were described [Supplementary data (Table I)], with fatigue, breathlessness and memory problems being the most common at present. These symptoms have been reported previously.^6^ ^,^ ^7^ ^,^ ^19^ ^-^ ^22^ A limitation of this study is that the variants causing Long COVID are unknown. In most cases, the COVID-19 diagnosis was made in other public health facility without sequencing the virus genome, and then the patients were referred to the HUPE outpatient clinic.

Along with the difficulties in determining a clear-cut test for the diagnosis of Long COVID, there is also debate about who is more susceptible to Long COVID. In this study, developing Long COVID was not associated with the severity of COVID-19 in the cohort comprised of middle-aged individuals. It is possible that the risk factors for Long COVID are related to female gender, previous medical co-morbidities and the virus genome variants.^33^ ^,^ ^34^ There was no gender bias in the recruitment phase of this work; however, it is clear that females were in the majority (31/34). One possible explanation is that female hormones played a role in maintaining the over-inflammation seen in the acute phase of Long COVID.^35^

In the majority of patients, blood counts return to normal parameters within a few days after the acute phase has ended.^35^ However, a minority of patients may experience changes in their leukograms. In this study, six out of 34 patients had changes in their white blood cell counts. Four of them had leucocytosis at the expense of neutrophils and one had leukopenia due to a decrease in neutrophils. One patient had an increased N/L ratio without any previous report of chronic illness. One possible explanation is that Long COVID triggered these changes or that the patients could be carriers of another underlying infectious disease.

In Brazil, the vaccination campaign against SARS-COV-2 began in January 2021. The Brazilian health regulatory agency (ANVISA) initially approved four vaccines: CoronaVac, Pfizer-BioNTech, Oxford-AstraZeneca, and Janssen. Moderna was also recently approved by ANVISA.^36^ The vaccination schedule was initially homologous and then became heterologous. The aim of the vaccination was to reduce the risk of developing severe COVID-19 symptoms. However, the vaccination may partially protect against the effects of Long COVID or mitigate symptoms.^37^ After vaccination, especially during the Omicron variant period, the risk of developing Long COVID was reduced but still exists.^38^ However, in this study, most (31/34) of the Long COVID patients surveyed reported having received at least two doses but still continue to suffer from Long COVID symptoms.

An Italian study reported a significant shortening of LTL, along with other age-related changes, in post-COVID-19 patients, as determined by pyrosequencing of defined CpG islands.^39^ In contrast, in this study it was demonstrated that telomeres (LTL) are longer in the Long COVID patient group in comparison to the controls. This observation was confirmed by the re-evaluation of LTL in a subgroup of nine patients 15 months later. Differences (genetic, clinical, and environmental) in the study populations and in the methods of analysis could explain these contradictory results. Another possible explanation is that the successive emergence of SARS-CoV-2 variants, along with vaccination, has altered the severity of COVID-19 and the resulting Long COVID.

Even taking into account the small number of Long COVID patients and controls recruited for the telomerase activity assay, the finding of telomerase activity in this group of Long COVID patients was surprising. Reactivation of telomerase is the most likely explanation for the elongation of telomeres in Long COVID patients. In normal blood cells, telomerase activity is low and consequently insufficient to prevent telomere shortening throughout life.^14^ The regulation of telomerase activity/hTERT expression involves genetic and epigenetic factors.^40^ ^,^ ^41^ ^,^ ^42^ In the Long COVID scenario, it is quite possible that the persistently high concentration of cytokines plays a role in the reactivation of telomerase, since cytokines can promote the expression of hTERT, the catalytic unit of telomerase.^14^ ^,^ ^43^ ^,^ ^44^ It is worth noting that higher cytokine levels and longer telomeres were observed in ageing elite athletes.^45^

Another limitation of this work is that there are no data on LTL and telomerase activity before and during acute COVID-19 symptoms in the Long COVID patients. It cannot be ruled out that the telomeres of these patients were already elongated before COVID-19. Furthermore, it is not known whether the medications administered or the study participants’ previous chronic illnesses triggered changes in LTL.

The health consequences caused by the elongation of telomeres and telomerase activity observed in Long COVID patients are completely unknown. It will be necessary to conduct further studies and monitor Long COVID patients to determine if future health problems are due to telomerase activity and elongated telomeres. In conclusion, the full underlying biology of Long COVID needs to be uncovered and the role of TL should be considered. Telomerase reactivation could be part of this complex scenario and likely caused extended telomeres.
