# Supplementary material for: Leukocyte telomere length and telomerase activity in Long COVID patients from Rio de Janeiro, Brazil
Source: Mem Inst Oswaldo Cruz. 2025 Apr 11;120:e240129. doi: 10.1590/0074-02760240129 (PMC11984962; doi:10.1590/0074-02760240129)
Supplement: Supplementary file 1 [file 1678-8060-mioc-120-e240129-s.pdf]

Hbg and Tel amplification curves

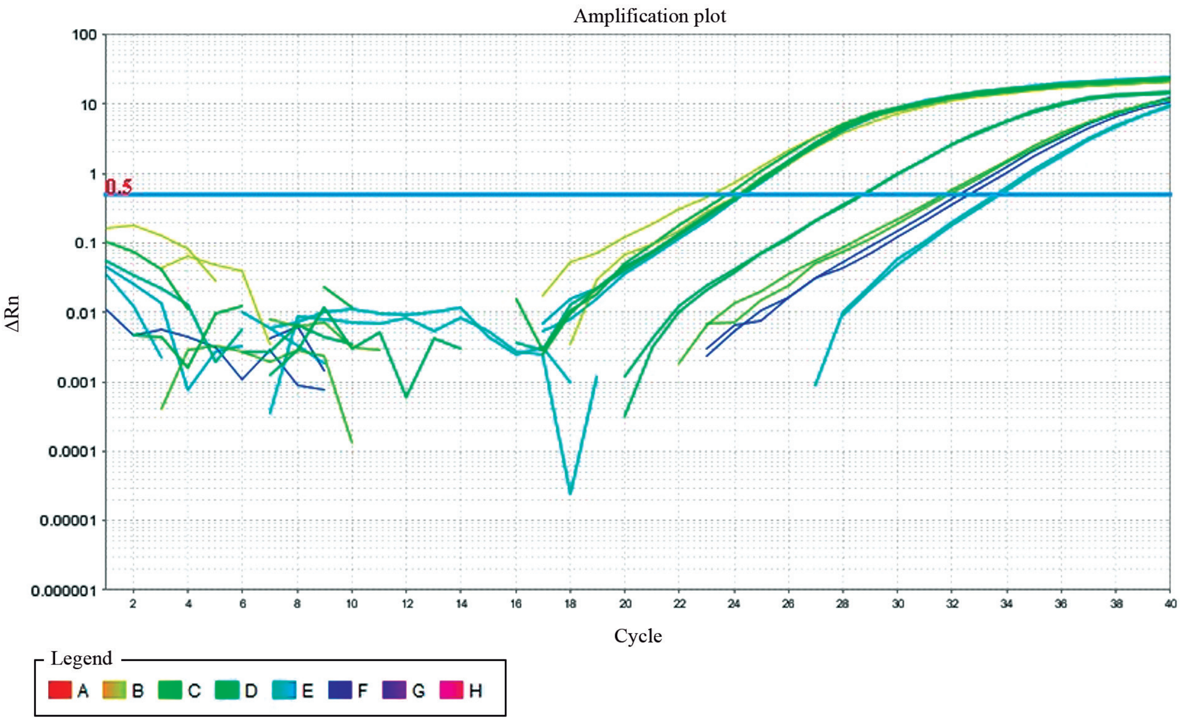

Hbg - Ct approximately 20. Tel - Ct > 26

Fig. 1: *Hbg* gene - Ct approximately 20. Tel Ct > 26.

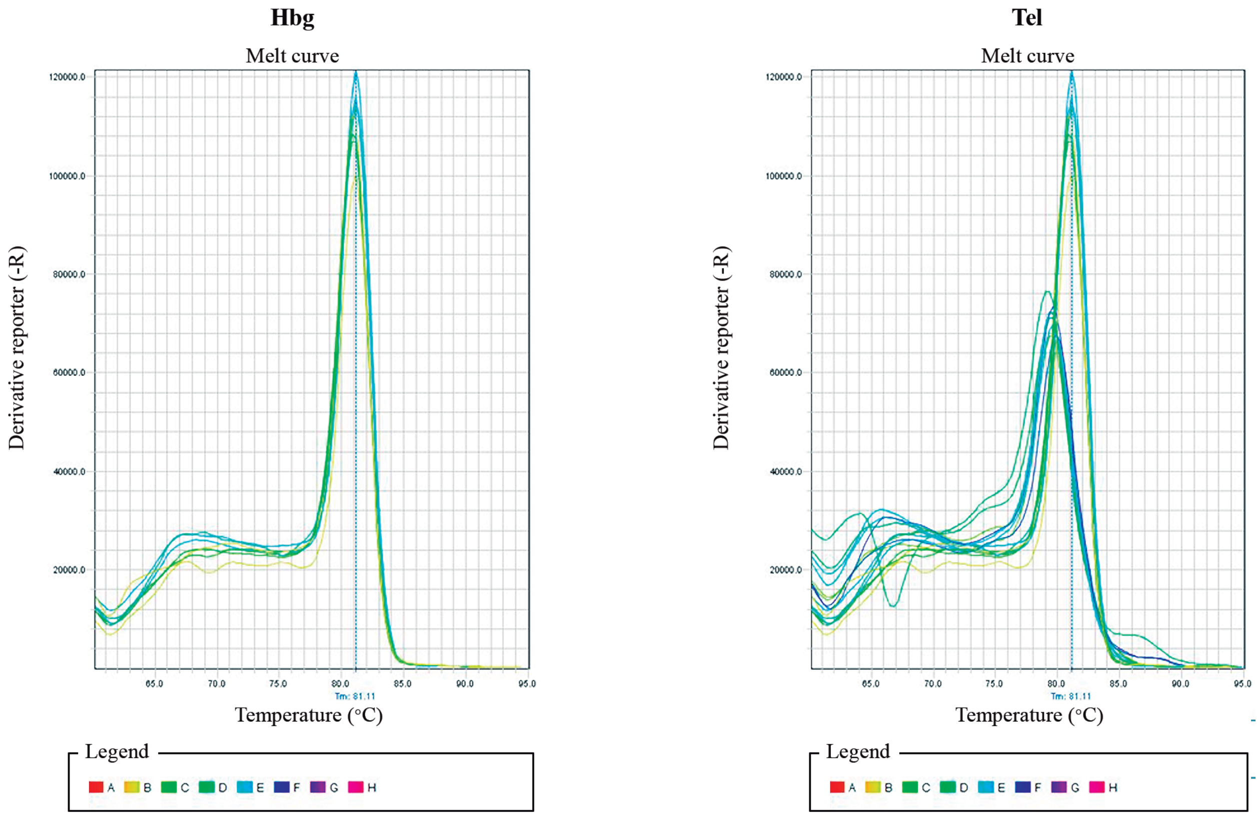

Fig. 2: melting curves analysis from qPCR for *Hbg* gene (Tm = 81.11ºC) and Tel (Tm = 81.11ºC).

CC BY

TABLE I

Symptoms in acute and post-COVID infection

Patient	Symptoms in acute infection	Symptoms in Long COVID
1	1, 3, 5, 7, **8**, **15**, 23, 31	**8**, **15**, 25
2	1, 2, **3**, **5**, 6, **7**, 8, 10, 13, 23	**3**, **5**, **7**, 26, 27, 29
3	3, **5**	4, 5, 29, 37, 41, 42, 43
4	3, **4**, **5**	**4**, **5**, 15, 19, 20, 26, 29
5	1, 2, **3**, **4**, **6**, 7, 8, 9, 10, 11, **12**, 15, 19, 22, 23	**3**, **4**, 5, **6**, **12**, 13, 19, 20, 24, 25, 26, 27, 28, 37, 38
6	1, 2, 3, **4**, **5**, 6, 7, 8, 9, 11, 12, 13, 19, 21, 22, 20, **28**, 31	**4**, **5**, 15, 24, 25, 26, 27, **28**, 38, 39, 40
7	1, 4, 6, **7**, **8**, 13, **15**	**7**, **8**, 12, **15**, 19, 26
8	1, 2, 3, 4, 5, 7, 8, 12, 13, **15**, **20**, 23, 28	**15**, 19, **20**, 24, 26, 27, 40
9	1, 2, 3, **4**, **5**, 6, **7**, **8**, 9, 10, 11, 13, **15**, 17, **19**, 21, 22, 23, **28**, 31	**4**, **5**, **7**, **8**, **15**, **19**, 24, 25, 26, **28**, 29, 37, 38, 39, 40, 41, 42
10	1, 3, **4**, **5**, **6**, 7, 8, 9, 10, 11, 12, **13**, **15**, **19**, **20**, **28**	**4**, **5**, **6**, **13**, **15**, **19**, **20**, 26, **28**, 29, 38, 39, 40, 42
11	**3**, **4**, **6**, **7**, **8**, **13**, **15**, 21, 22, **28**	**3**, **4**, **6**, **7**, **8**, **13**, **15**, 19, 25, **28**, 39, 41
12	4, 5	7
13	1, 3, **4**, 5, 6, 9, 10, 13, **15**, **17**, **19**, 28, 31	**4**, 12, **15**, **17**, **19**, 26
14	1, **3**, **4**, **6**, **7**, **8**, 9, **13**, **15**, **19**, **20**, **29**	**3**, **4**, **6**, **7**, **8**, **13**, **15**, **19**, **20**, 25, **29**
15	3, **4**, **5**, 9, 15, 19, 20, 31	**4**, **5**
16	1, 6, 7, 8	18, 42
17	1, 3, **4**, **5**, 6, 9, 10,	**4**, **5**
18	1, 3, **4**, 5, 6, 9, 10, **12**, **13**, **15**, **19**, 31	**4**, **12**, **13**, **15**, **19**, 20, 29
19	1, 2, **4**, **15**, **20**	**4**, **15**, **20**, 26, 29
20	1, **3**, **4**, 5, **8**, 13, **19**, 21, 28	**3**, **4**, **8**, **19**, 25, 27, 29, 39, 42
21	1, 2, **3**, **4**, **5**, 6, 10, 13, **15**, 22, **23**	**3**, **4**, **5**, 12, **15**, 19, 20, **23**, 24, 26, 27
22	3, **5**	4, **5**
23	1, 4	19, 20, 24, 29, 37, 38, 39, 41
24	1, 2, 3, **4**, 5, **6**, 7, 8, 9, 10, **15**, **20**, 22, 23, 28	**4**, **6**, **15**, **20**, 24, 26, 37, 38, 39, 40, 41
25	1, 2, 3, **4**, **5**, **6**, **7**, **8**, 9, 10, 11, **12**, **13**, **15**, 17, **19**, 21, 23, **28**	**4**, **5**, **6**, **7**, **8**, **12**, **13**, **15**, **19**, 20, 24, 25, 26, **28**, 37, 38, 39, 40, 41, 42
26	1, **5**, **19**, **20**	4, **5**, 13, **19**, **20**, 24, 26, 39, 40, 41, 42
27	1, 2, **3**, **5**, 6, **7**, 8, 10, 13, 23	**3**, **5**, **7**, 26, 27, 29
28	1, 2, 3, **4**, **5**, 6, 7, 8, 9, 11, 12, 13, **15**, 19, 20, 21, 22, **28**, 31	**4**, **5**, **15**, 24, 25, 26, **28**, 38, 39, 40
29	1, 2, 3, **4**, **5**, **6**, **13**, **15**, **19**, 22, **28**, 31	**4**, **5**, **6**, 7, **13**, **15**, **19**, 24, 26, 29, **28**, 37, 38, 39, 40, 41, 42
30	1, 3, **4**, **6**, 7, 8, 10, **15**, 18, **19**, 22, 31	**4**, 5, **6**, **15**, **19**, 40
31	1, 2, 3, **4**, **6**, 7, 8, 9, 12, 13, **15**, **19**, 23, **28**	**4**, 5, **6**, **15**, **19**, **28**, 29, 37, 39
32	**6**, 9, 10	4, 5, **6**, 15, 19, 28, 29, 36, 38
33	1, 4, **6**, 7, 8, 22	**6**, 15, 24, 25, 27
34	3, 6, 7, 8, 9, **10**, 28, 31	**10**, 24, 27, 29, 38, 39

1 - fever; 2 - chills; 3 - cough; 4 - fatigue; 5 - dyspnoea; 6 - headache; 7 - anosmia/ hyposmia; 8 - ageusia/hypoageusia; 9 - sore throat; 10 - nasal congestion; 11 - conjunctival congestion; 12 - palpitation; 13 - chest pain; 14 - earache; 15 - body ache; 16 - back pain; 17 - abdominal pain; 18 - leg pain; 19 - arthralgia; 20 - myalgia; 21 - skin rash; 22 - nausea/vomiting; 23 - loss of appetite; 24 - mood changes; 25 - memory deficiencies; 26 - anxiety; 27 - depression; 28 - voice change; 29 - hair loss; 30 - diarrhoea; 31 - bleeding when evacuating; 32 - cold; 33 - skin itching; 34 - change in blood pressure; 35 - nasal runny nose; 36 - insomnia; 37 - vertigo; 38 - dizziness; 39 - excessive sweating; 40 - swelling; 41 - impaired mobility; 42 - numbness in lower limbs. Numbers in bold - symptoms that were present in the acute phase and continued in Long COVID.

TABLE II

White blood count of Long COVID patients

Patient	Leu (3,600-11,000/mm³)	N (40-78%)	N (1,700-8,200/mm³)	L (20-50%)	L (1,000-4,500/mm³)	Ratio N/L
1	8,510	61	5,191	32	2,732	1.90
2	9,020	70	6,314	25	2,255	2.80
3	7,360	63	4,636	31	2,281	2.03
4	6,170	53	3,270	42	2,591	1.26
5	9,520	53	5,045	40	3,808	1.32
6	6,500	63	4,095	29	1,885	2.17
7	**14,760**	67	**9,889**	27	3,985	2.48
8	8,700	55	4,785	38	3,306	1.44
9	6,980	66	4,606	28	1,954	2.35
10	8,280	58	4,802	33	2,732	1.75
11	3,850	**82**	3,157	**14**	**539**	**5.85**
12	7,230	61	4,627	30	2,169	2.13
13	4,330	45	1,948	44	1,905	1.02
14	**3,500**	42	**1,479**	46	1,610	0.91
15	5,980	56	3,348	36	2,152	1.55
16	5,780	63	3,641	31	1,791	2.03
17	6,570	56	3,679	37	2,430	1.51
18	5,550	60	3,330	31	1,720	1.93
19	3,760	63	2,368	31	1,165	2.03
20	8,120	65	5,278	30	2,436	2.16
21	**12,220**	68	**8,309**	24	2,932	2.83
22	6,540	56	3,662	36	2,354	1.55
23	5,440	49	2,665	42	2,284	1.16
24	7,750	62	4,805	31	2,402	2.00
25	6,650	66	4,389	28	1,862	2.35
26	11,410	80	9,128	15	1,711	**5.33**
27	10,960	59	6,466	31	3,397	1.90
28	7,100	62	4,402	31	2,201	2.00
29	7,820	56	4,379	33	2,580	1.69
30	5,430	57	3,095	34	1,846	1.67
31	**18,040**	**84**	**15,153**	**9**	1,623	**9.33**
32	4,720	65	3,068	28	1,321	2.32
33	4,580	57	2,610	35	1,603	1.62
34	4,240	58	2,458	34	1,441	1.70

Leu: leukocytes; N: neutrophils; L: lymphocytes. Values in bold are the alterations.
